# Machine learning and bioinformatics-based identification of mitophagy-related diagnostic biomarkers in bronchiolitis obliterans

**DOI:** 10.1097/MD.0000000000047672

**Published:** 2026-02-13

**Authors:** Ying Wang, Wenbin Peng

**Affiliations:** a Pediatric Department, Pinghu Maternal and Child Health Care Hospital, Pinghu City, Zhejiang Province, China; b Gynaecology and Obstetrics Department, Pinghu Maternal and Child Health Care Hospital, Pinghu City, Zhejiang Province, China.

**Keywords:** bronchiolitis obliterans (BO), diagnose, machine learning, mitophagy

## Abstract

This study aimed to explore the molecular mechanisms associated with mitophagy in BO and identified mitophagy-associated BO diagnostic genes. Using Gene Expression Omnibus database data, differentially expressed genes in BO patients vs controls were analyzed via Gene Ontology enrichment. Algorithms like Boruta, least absolute shrinkage and selection operator, and Random Forest screened BO-specific genes. Mitophagy genes were sourced from PathCards and correlated with BO-specific genes via single-sample gene set enrichment analysis (ssGSEA). Receiver operating characteristic curves evaluated the diagnostic performance of these genes. Two hundred and six differentially expressed genes were identified, in which immune-related pathways such as the B-cell receptor signaling pathway and lymphocyte differentiation were significantly enriched. Machine learning screening yielded 30 BO signature genes, among which KLRC3 and CD36 were significantly correlated with ssGSEA enrichment score of the mitophagy gene sets. Receiver operating characteristic analysis confirmed their diagnostic value with AUCs of 0.648 and 0.640, respectively. This finding indicated that KLRC3 and CD36 are not only significantly correlated with ssGSEA enrichment score of the mitophagy gene sets but also have diagnostic value for BO.

## 1. Introduction

Bronchiolitis obliterans (BO) is a rare but serious respiratory disease characterized by chronic airflow limitation, mainly affecting the small airways, and is common in patients following infection or exposure to harmful substances.^[1]^ Postinfectious occlusive bronchiolitis obliterans is particularly common in children. Pediatric pneumonia is an important contributor to BO, with adenoviral infections accounting for a significant proportion of BO cases in children, and other pathogens such as *Mycoplasma pneumonia*, measles virus, respiratory syncytial virus and influenza virus are also thought to be involved in the development of postinfectious bronchiolitis obliterans.^[2]^ The direct link between pediatric pneumonia and BO is further supported by studies showing that adenoviral infections occur in approximately 5.7% to 11.0% of cases of community-acquired pneumonia in children.^[3]^ The inflammatory response and tissue repair process following infection may lead to airway remodeling and obstruction, which can trigger the onset and progression of BO.

It has been suggested that abnormal mitochondrial function is involved in the pathogenesis of BO.^[4]^ Mitophagy is a specific autophagic process that removes damaged mitochondria to maintain normal cell function and health.^[5-7]^ Abnormalities in the mitophagy pathway have been associated with developing several lung diseases.^[8,9]^ For example, mutations in the autophagy regulator ATG16L1 lead to protein instability that affects mitochondrial function in monocyte-derived macrophages,^[10]^ which may influence the onset and development of BO. Also, oxidative stress and metabolic changes in monocyte-derived alveolar macrophages due to mitochondrial damage are closely related to the development of BO.^[11]^ These findings suggest that mitophagy plays an important role in the pathological progression of BO, but this aspect has not been fully investigated.

This study proposes to explore mitophagy-related biomarkers in occlusive bronchiectasis by identifying diagnostic biomarkers associated with BO from high-throughput gene expression data using machine learning and bioinformatics to provide new strategies for early diagnosis and personalized treatment of BO.

## 2. Materials and methods

### 2.1. Gene expression dataset

GSE94557 was obtained from the Gene Expression Omnibus database (https://www.ncbi.nlm.nih.gov/geo/). This dataset represents the use of Agilent SurePrint G3 Human Gene Expression v3 8 × 60 K microarrays from 80 patients (40 stable and 40 bronchiolitis obliterans syndrome) from gene expression analysis of blood samples collected in vivo from PAXgene.^[12]^

### 2.2. Identification of BO-associated DEGs

First, we standardized and normalized the 2 data sets in the training set to eliminate experimental bias and data noise. The 2 data sets were compared using the *edgeR R* package to screen for differentially expressed genes (DEGs). To capture more DEGs, especially those with small changes that may be biologically significant. We defined the screening criteria as |log_2_ fold change| > 0.2 and FDR value < 0.05. Through this process, we identified the set of DEGs in patients with BO, which provided a list of candidate genes for subsequent analyses. The screened differential genes were subjected to Gene Ontology (GO) enrichment analysis to explore their biological functions and potential signaling pathways. GO analysis included 3 aspects: molecular function, cellular component and biological process.

### 2.3. Machine learning to select feature genes

Based on the BO differential genes identified above, we used 3 machine learning methods, Boruta, least absolute shrinkage and selection operator (LASSO), and Random Forest, to select the feature genes most relevant to BO diagnosis.

#### 2.3.1. Boruta

The Boruta algorithm is an extension of RF for feature selection that aims to identify the most important variables for the prediction task from many features. Unlike traditional feature selection methods, Boruta uses the original features to assess their importance and introduces “shadow features” to determine the relative importance of the features, thus more accurately selecting the features significantly related to the target variable.^[13]^ The Boruta algorithm assigns an importance score to each feature, which is calculated based on the performance of the RF model in feature prediction. The importance of features is usually presented in the form of a Z-score. Boruta can also make decisions to discriminate features as “Confirmed,” “Rejected,” and “Tentative.” In this study, we set the maximum number of iterations of Boruta to 100 and retained the tentative features.

#### 2.3.2. LASSO

LASSO is a regularization method for regression analysis, especially used for feature selection and model compression in high-dimensional data. LASSO achieves feature selection by applying an L1 penalty (i.e., the sum of the absolute values of the regression coefficients) on the regression coefficients, compressing certain regression coefficients to 0 and thus acting as a feature selector. The advantages of the LASSO method are that it is effective in reducing the problem of multicollinearity, improving the interpretability of the model, and preventing overfitting.^[14]^ LASSO regression ultimately produces 2 results: Nonzero coefficients: the LASSO retains the features that have a significant relationship with the predicted target variable and the regression coefficients of these features are shrunk to the appropriate values by regularization and 0 coefficients: the LASSO shrinks those features that are weakly associated with the target variable to 0. features that are weakly associated with the target variable are contracted to 0, thus enabling feature selection. The LASSO algorithm usually uses cross-validation to select the appropriate regularization parameter (λ). λ controls the strength of the L1 penalty term: when the value of λ is large, the regularization effect is enhanced, and more regression coefficients are contracted to 0, leading to a more simplified model, but possibly at the expense of some prediction accuracy. In this study we chose lambda.min (typically the value of λ at which the cross-validated mean square error is smallest) for inclusion in the model and excluded genes with 0 coefficients.

#### 2.3.3. Random Forest

RF is an integrated learning method based on decision trees, which improves the accuracy and stability of a model by constructing multiple decision trees and combining their predictions. Each tree is trained independently on a randomly selected subset of samples and features from the training data, and the final output is determined by majority voting (classification task) or averaging (regression task).^[15]^ During tree construction, the RF calculates the total contribution of each feature to the Gini index (classification task) or variance reduction (regression task) across all split nodes and normalizes it to derive an importance score that measures the feature’s contribution to model prediction. In general importance scores > 0 indicate a positive contribution and importance scores < 0 indicate a negative contribution. In this study, we screened the genes with importance score > 0 by RF.

### 2.4. Identification of BO and mitophagy-related signature genes

We obtained the mitophagy gene set from the PathCards database (https://pathcards.genecards.org), which included ATG12, ATG5, ATG9A, CSNK2A1, CSNK2A2, CSNK2B, FUNDC1, MAP1LC3A, MAP1LC3B, MFN1, MFN2, MTERF3, OPTN, PEDS1-UBE2V1, PGAM5, PINK1, PRKN’, ’RPS27A, SQSTM1, SRC, TBK1, TOMM20, TOMM22, TOMM40, TOMM5, TOMM6, TOMM7, TOMM70, UBA52, UBB, UBC, UBE2D2, UBE2D3, UBE2L3, UBE2N, UBE2V1, ULK1, VDAC1, VDAC2, and VDAC3. Since MTERF3, PEDS1-UBE2V1, PGAM5, PRKN, TOMM40, and TOMM70 were in the gene expression data quality control process were excluded, we therefore analyzed the other 34 genes in this section. We intersected these genes with the previously selected set of BO signature genes and found no common genes. Therefore, we further plotted the correlation heatmap by calculating the correlation (Pearson correlation coefficient) between the BO signature genes and the mitophagy gene set, setting *P* < .05 as the criterion for statistical significance. To further select the most relevant genes for mitophagy, we calculated the enrichment of the mitophagy gene set by the single-sample gene set enrichment analysis (ssGSEA) method and performed Pearson correlation analyses between the enrichment and the BO feature genes.

### 2.5. Diagnostic performance analysis of signature genes

To assess the performance of the screened feature genes in BO diagnosis, we plotted receiver operating characteristic (ROC) curves. The diagnostic performance of each feature gene in the validation group was assessed by calculating the area under the curve (AUC) value, sensitivity, specificity, etc. The closer the AUC value was to 1, the stronger the diagnostic ability of the gene. All statistical analyses were performed using R 4.4.2, and *P* < .05 was considered statistically significant. This study was approved by the Ethics Committee of Pinghu Maternal and Child Health Care Hospital.

## 3. Results

### 3.1. DEGs in BO

**Figure 1.**
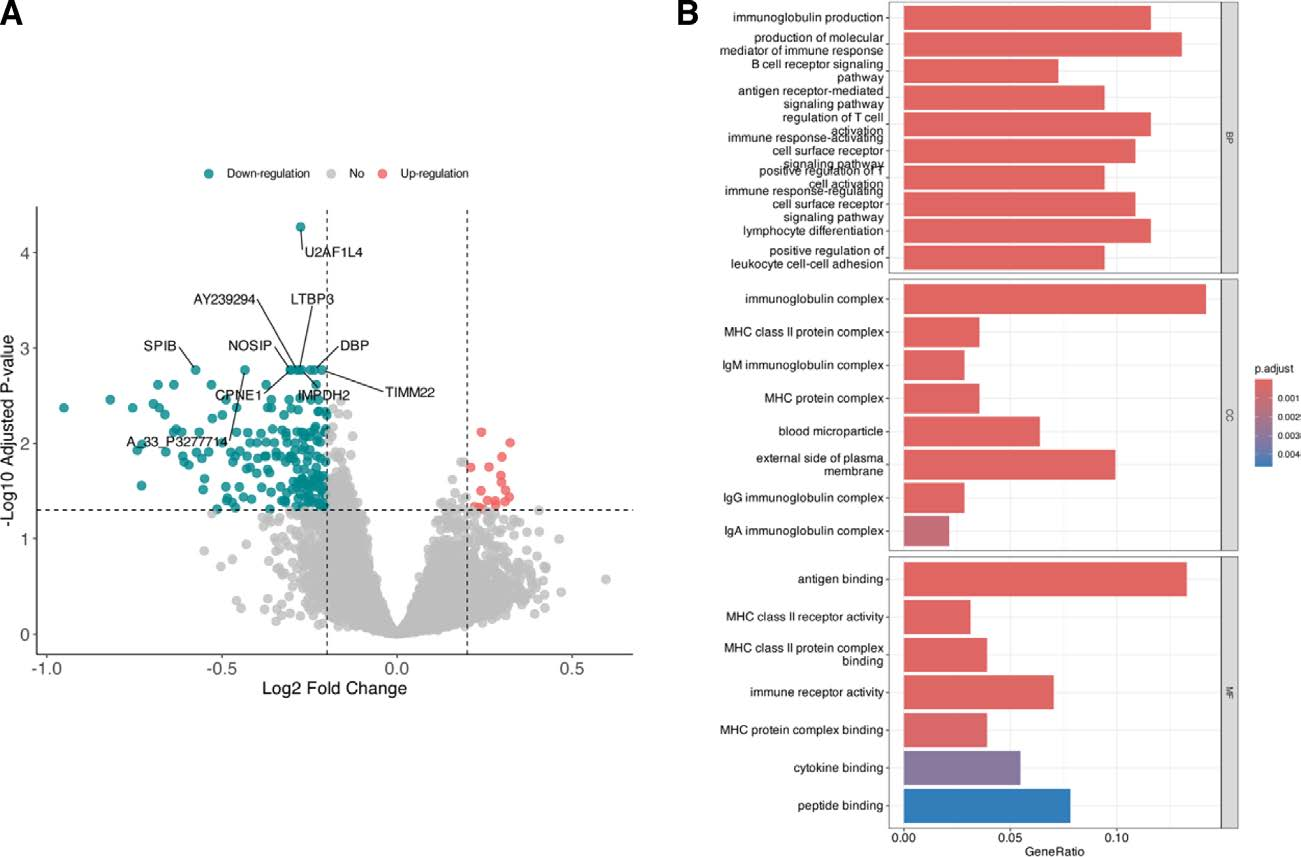
Volcano plot of DEGs and results of GO enrichment analysis. (A) Volcano plot. (B) The results of GO enrichment analysis. DEG = differentially expressed gene, GO = Gene Ontology

This study used a whole blood cell large-scale gene expression microarray (GSE94557) to analyze gene expression between BO patients and controls differentially. A total of 206 significantly DEGs were screened, of which 17 were significantly upregulated and 189 were significantly downregulated (Fig. 1 A). The 10 most significantly DEGs were LIMS2, HLA-DQA1, TRBV6-6, REC8, CD3E, NOLC1, BFSP1, HL A-DQA2, IL23A, and OSBPL5. The results of the GO enrichment analysis showed that DEGs were highly enriched in the immune response, antigen recognition, and inflammation regulation, as shown in Figure 1 B. In the DEGs screening process, the raw gene expression data were first normalized to remove any batch effects or experimental bias. This standardization was essential to ensure that any observed gene expression differences were biological rather than technical artifacts. After normalization, the data were analyzed using the edgeR R package to identify genes that were significantly differentially expressed between patients with BO and control subjects. Genes were considered differentially expressed if their |log_2_ fold change| was >0.2 and the false discovery rate value was <0.05. This stringent criterion enabled the identification of 206 DEGs, which were further investigated to understand their potential roles in BO pathology.

### 3.2. Screening for BO BO-associated signature genes

Subsequently, based on the above set of differential genes, we used machine learning methods such as Boruta, LASSO and RF to screen the genes with the most diagnostic value for BO.

**Figure 2.**
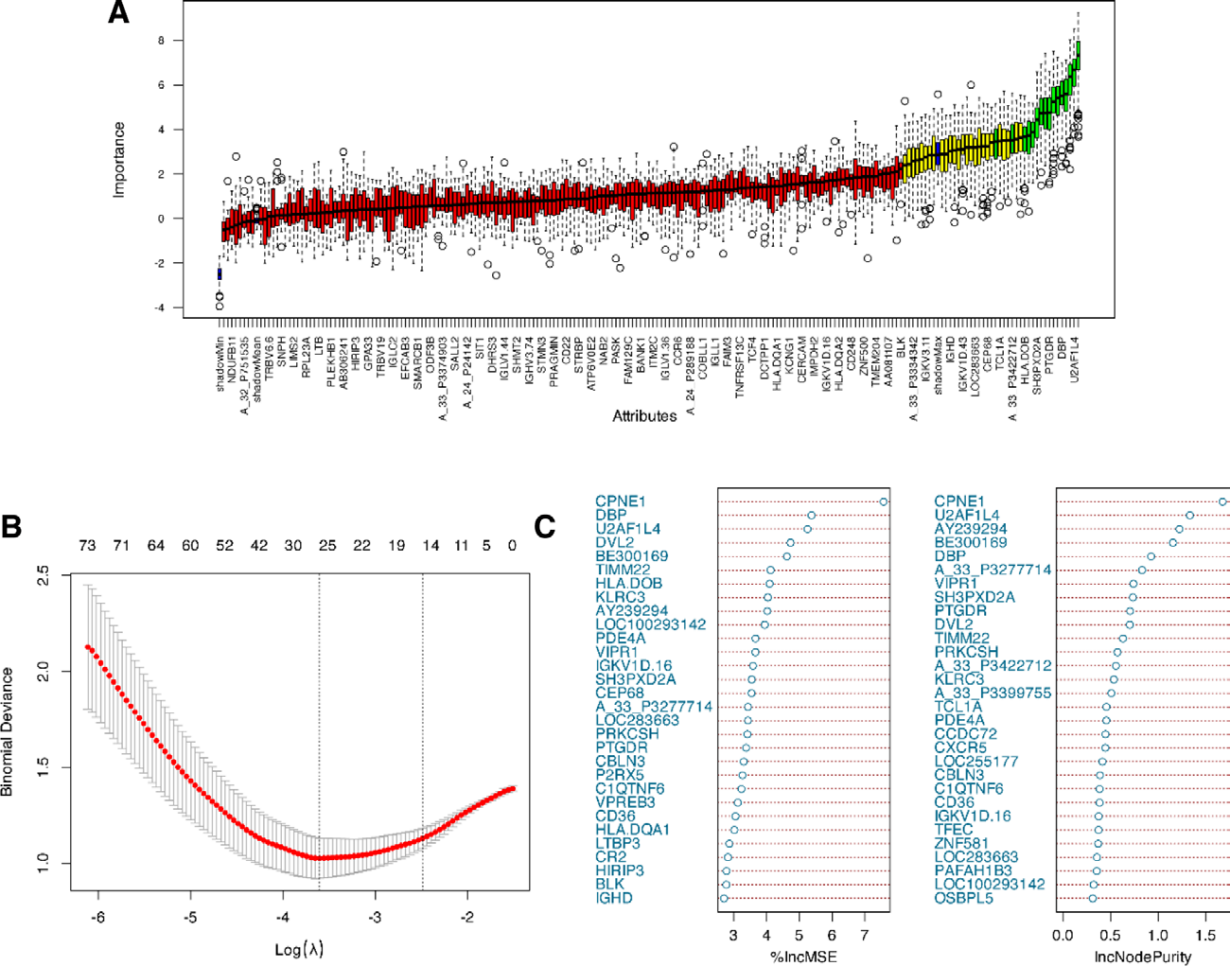
Machine learning screening for BO BO-associated signature genes. (A) For screening results for Boruta, the *x*-axis is the gene name, and the *y*-axis is the important gene feature. (B) LASSO regression cross-validation to select the λ. (C) Feature importance ranking under the RF algorithm. BO = bronchiolitis obliterans, LASSO = least absolute shrinkage and selection operator, RF = Random Forest.

The Boruta algorithm for feature selection was set to have a maximum number of iterations of 100, and 51 key genes were screened (Fig. 2A).

In the LASSO regression model, the x-axis in the graph represents the logarithmic value of the regularization parameter (Log(λ)), which controls the strength of feature selection in the model. The smaller the value, the weaker the constraint and the more features are retained. The *Y*-axis represents the bias of the model (binomial deviance), and the smaller it is, the better the model fit. The red dots and gray error bars represent the mean deviation corresponding to each λ and its standard error, and the gray error bars reflect the uncertainty range. The left vertical line indicates the optimal λ value (so that the deviation is minimal), and the right vertical line indicates the λ with good generalization performance. We set the λ with the smallest deviation from the λ value (0.02724898) and selected the genes with nonzero coefficients, and the results showed that 25 genes contributed to the classification task (Fig. 2B).

Furthermore, we used the Random Forest Model algorithm to rank the importance of the difference genes, and the left “%IncMSE” in the figure indicates the percentage contribution (% Increase in Mean Squared Error) of each variable to the mean square error boost in the Random Forest Model. The higher up the variable is, the more the variable contributes to the predictive performance of the model. If the increase in MSE is greater when a variable is removed, the more important the variable is. “IncNodePurity” indicates the cumulative contribution of each variable to the increase in Node Purity when divided into nodes. The higher the variable is, the better the classification ability of the variable is. Finally, RF filtered out a total of 163 genes with positive contribution (importance score > 0), and the top 30 genes in terms of importance are shown in the figure (Fig. 2C).

Finally, we took the intersection of the genes screened by the 3 machine learning filters, resulting in a total of 30 feature genes: U2AF1L4, CD36, DBP, KLRC3, PTGDR, CPNE1, PDE4A, SH3PXD2A, SPIB, BE300169, VIPR1, TIMM22, CBLN3, and A_33_P3768930, A_33_P3399755.

### 3.3. Identification of signature genes associated with mitophagy in BO

**Figure 3.**
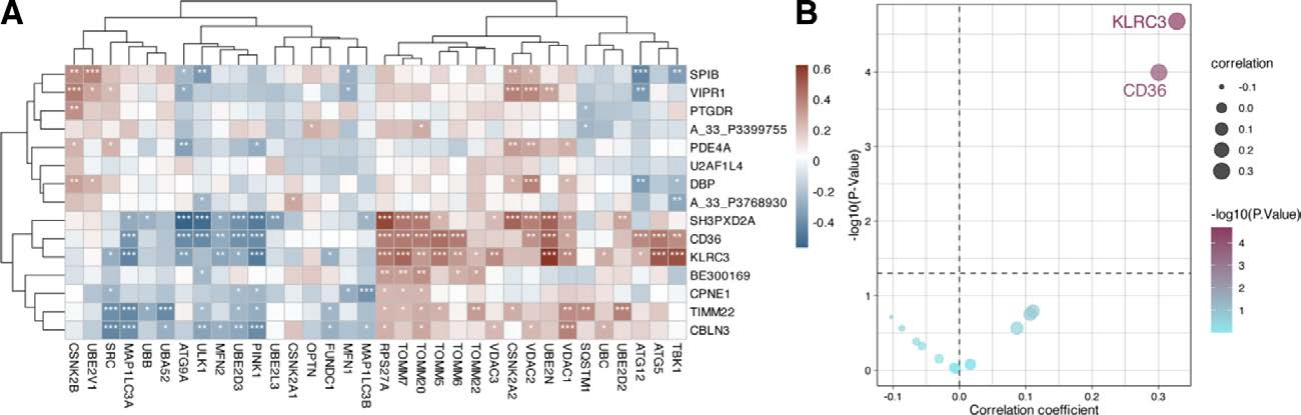
Relationship of BO signature genes to mitophagy-related genes. (A) Relationship of BO signature genes to various mitophagy-related genes. * *P* < .05, ** *P* < .01, *** *P* < .01 (B) Relationship between BO signature genes and enrichment scores of autophagy genes in each mitochondrion. The *x*-axis is the correlation coefficient; the *y*-axis is -log (*P*-value), and the horizontal dashed line corresponds to a value of −log (0.05), above which genes significantly correlate with the ssGSEA enrichment score of the mitophagy gene set. BO = bronchiolitis obliterans, ssGSEA = single-sample gene set enrichment analysis.

Subsequently, to find the characteristic genes associated with mitophagy in BO. We obtained the mitophagy gene set in the PathCards database (https://pathcards.genecards.org). The mitophagy gene set in the PathCards database contains 40 genes, and 34 mitophagy genes were used for the analyses after removing genes that were rejected during the quality control stage in the samples. We first used correlation analysis to screen for BO signature genes associated with mitophagy genes (*P* < .05; Fig. 3A). Due to the large number of feature genes associated with mitophagy, we then calculated the enrichment scores of mitophagy genes using the ssGSEA method. We analyzed the correlation between BO feature genes and them (Fig. 3B). The results showed that KLRC3 and CD36 genes were significantly correlated with ssGSEA enrichment score of the mitophagy gene sets.

### 3.4. Screening for BO BO-associated signature genes

**Figure 4.**
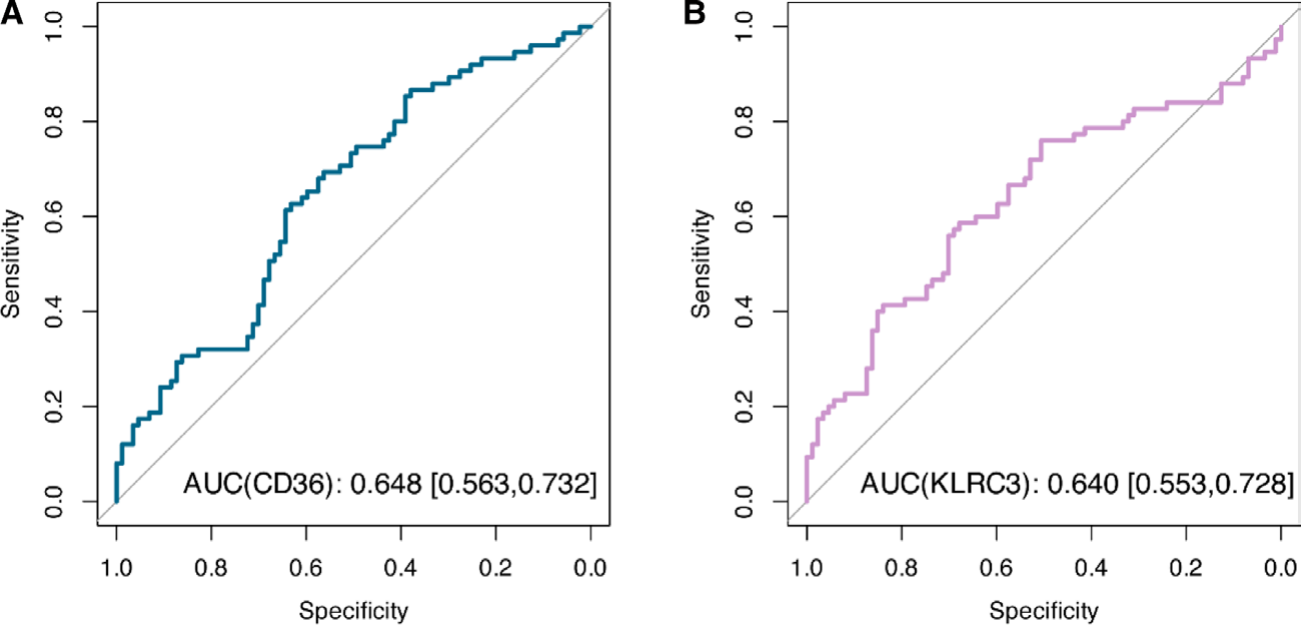
Results of ROC curve analysis of CD36 (A) and KLRC3 (B) for the diagnosis of BO. AUC = area under the curve, ROC = receiver operating characteristic.

We used ROC curve analysis to perform KLRC3 and CD36 in the BO diagnostic task. ROC curves were plotted by calculating the sensitivity and specificity of KLRC3 and CD36 at different thresholds. The AUC values for KLRC3 and CD36 were 0.648 (95% 0.563–0.732) and 0.640 (95% 0.553–0.728), respectively (Fig. 4). This indicates that KLRC3 and CD36 genes have some BO diagnostic properties.

## 4. Discussions

This study’s 206 DEGs identified by gene expression analysis reflect significant abnormalities in BO patients’ immune response and tissue remodeling. GO enrichment analysis showed that these genes were significantly enriched in BPs such as immune regulation and lymphocyte differentiation. This demonstrated the key role of inflammatory response and immune imbalance in the development and progression of BO. Infection is an important causative agent of BO, especially in children, and adenovirus infection is considered one of the major causes of BO. The inflammatory response triggered by infection can lead to small airway epithelial damage and fibrosis, triggering the onset and progression of BO.^[16,17]^ In addition, studies have shown that immune-related processes, such as immunoglobulin production, regulation of B-cell receptor signaling pathways and T-cell activation, are significantly abnormal in patients with BO, suggesting that immune imbalance may contribute to the progression of BO.^[18,19]^

We screened 2 BO diagnostic genes associated with mitophagy, CD36 and KLRC3, by machine learning algorithms and correlation analysis with ssGSEA enrichment score of the mitophagy gene sets. CD36 is a multifunctional transmembrane glycoprotein widely expressed in macrophages, endothelial cells, and various other cell types and plays a key role in lipid metabolism, inflammatory response, and oxidative stress.^[20-22]^ In recent years, it has been found that CD36 has an important role in mitophagy.^[23]^ Mitophagy is a key mechanism for cellular clearance of damaged mitochondria, and it is important in maintaining cellular homeostasis and reducing oxidative stress.^[24,25]^ Studies have shown that significant oxidative stress and immune disorders are present in patients with BO and that CD36 may indirectly affect mitochondrial function and autophagy processes by regulating lipid uptake and the accumulation of lipid oxidation products.^[26-28]^ Thus, CD36 may play a key role in these processes. The KLRC3 gene encodes a killer cell surface receptor expressed mainly in natural killer (NK) cells and some T cells.^[29]^ Studies have shown that mitophagy is important in maintaining the normal function of NK and T cells, ensuring that damaged mitochondria are removed to avoid cellular metabolic dysregulation and oxidative stress.^[30,31]^ Mitochondrial dysfunction and oxidative stress are common pathologies in patients with BO,^[4,32]^ and the KLRC3 gene may be indirectly involved in this process by regulating the activity of NK cells and T cells.^[33,34]^

## 5. Limitations

In this study, the results of ROC curve analysis showed that 2 mitophagy-related genes, KLRC3 and CD36 genes, had some discriminatory ability in BO diagnosis, suggesting their potential for clinical application. However, some limitations of this study still need to be stated. First, the performance of a single biomarker is still insufficient compared with the diagnostic needs of complex diseases. Future studies may combine multiple signature genes with clinical features and imaging data to develop a multidimensional diagnostic model to improve accuracy. In addition, the data in this study were mainly derived from publicly available databases and based on computational analyses only, lacking direct support from in vitro or in vivo experiments. Finally, although the study revealed a significant correlation between KLRC3 and CD36 and mitophagy, their specific functions and regulatory networks in BO have not been fully clarified.

## 6. Conclusions

Overall, this study provides promising insights into the role of mitophagy-related biomarkers, specifically KLRC3 and CD36, in the pathogenesis and diagnosis of BO. Although these findings suggest potential candidates for early disease diagnosis and targeted therapy, it is important to note that these biomarkers require further validation through in vitro and in vivo experiments. The clinical application of KLRC3 and CD36 as diagnostic tools will necessitate larger, multicenter studies and experimental verification before they can be considered for routine clinical use. This work opens a new avenue for investigating the molecular mechanisms of BO and offers a foundation for developing more precise diagnostic and therapeutic strategies in the future.

## Author contributions

**Conceptualization:** Ying Wang, Wenbin Peng.

**Data curation:** Ying Wang, Wenbin Peng.

**Formal analysis:** Ying Wang, Wenbin Peng.

**Funding acquisition:** Ying Wang, Wenbin Peng.

**Investigation:** Ying Wang, Wenbin Peng.

**Writing – original draft:** Ying Wang, Wenbin Peng.

**Writing – review & editing:** Ying Wang, Wenbin Peng.
